# Supplementary material for: Ion mobility-based sterolomics reveals spatially and temporally distinctive sterol lipids in the mouse brain
Source: Nat Commun. 2021 Jul 15;12:4343. doi: 10.1038/s41467-021-24672-x (PMC8282640; doi:10.1038/s41467-021-24672-x)
Supplement: Supplementary file 2 — Description of Additional Supplementary Files [file 41467_2021_24672_MOESM2_ESM.pdf]

Description of Additional Supplementary Files

Supplementary Data 1: The CCS values and differences of sterol isomers in derivatized and underivatized groups.

Supplementary Data 2: The peak resolutions (RS) in LC separation, IM separation and LC-IM based two-dimensional separation for sterol isomers.

Supplementary Data 3: The information of 97 sterol lipids in the standard ST library and 2068 sterol lipids in the extended ST Library.

Supplementary Data 4: The external validations of predicted CCS values and predicted RTs.

Supplementary Data 5: The four-dimensional identification of sterol lipids in plasma and liver tissue samples.

Supplementary Data 6: The four-dimensional identification of sterol lipids in brain tissue samples.

Supplementary Data 7: The distributions of shared and unique sterol lipids in ten brain regions.

Supplementary Data 8: The concentrations of sterol lipids measured in ten brain regions from mice at ages of 6 and 68 weeks.

Supplementary Data 9: The calibration curves of all 21 sterols identified in mouse brain samples.

Supplementary Data 10: The information of sterol lipids in clusters 1-5 in hierarchical clustering analysis.

Supplementary Data 11: The fold-changes of 102 dysregulated sterol lipids in ten mouse brain regions during aging.

Supplementary Data 12: The correlation information of dysregulated sterol lipids in ten brain regions during aging.
