# Supplementary material for: Ion mobility-based sterolomics reveals spatially and temporally distinctive sterol lipids in the mouse brain
Source: Nat Commun. 2021 Jul 15;12:4343. doi: 10.1038/s41467-021-24672-x (PMC8282640; doi:10.1038/s41467-021-24672-x)
Supplement: Supplementary file 3 — Supplementary Information [file 41467_2021_24672_MOESM3_ESM.pdf]

*Supplementary information for*

# Ion Mobility-based Sterolomics Reveals Spatially and Temporally Distinctive Sterol Lipids in Mouse Brain

*Tongzhou Li[1,2], Yandong Yin[1], Zhiwei Zhou[1,2], Jiaqian Qiu[1,2], Wenbin Liu[1], Xueting Zhang[1,2], Kaiwen He[1], Yuping Cai[1], and Zheng-Jiang Zhu[1,*]*

[1] Interdisciplinary Research Center on Biology and Chemistry, Shanghai Institute of Organic Chemistry, Chinese Academy of Sciences, Shanghai, 200032 P. R. China

[2] University of Chinese Academy of Sciences, Beijing, 100049 P. R. China

**Corresponding Author**

Correspondence should be addressed to Z.J.Z (jiangzhu@sioc.ac.cn)

## List of Supplementary Figures

**Supplementary Figure 1:** Examples for improved separation of sterol isomers with derivatization and IM-MS.

**Supplementary Figure 2:** The internal validations of CCS prediction and retention time prediction using the training data set.

**Supplementary Figure 3:** Validations of 21 sterol lipids in brain tissues using purchased chemical standards.

**Supplementary Figure 4:** Concentrations of secosterol-B, 24-isopropyl-cholesterol, and isofucostanol measured in brain regions.

**Supplementary Figure 5**: Concentration ranges of sterol lipids measured in ten brain regions.

**Supplementary Figure 6**: Percentage compositions of different sterol subclasses measured in ten brain regions.

**Supplementary Figure 7:** Concentration distributions of oxysterols, phytosterols and cholesterol derivatives in mouse brain regions.

**Supplementary Figure 8:** Amplitude changes of oxysterols, phytosterols and cholesterol derivatives measured in mouse brain regions.

**Supplementary Figure 9:** The estimation of false discovery rates (FDR) under different CCS matching tolerances using the extended ST library in mouse olfactory bulb region samples.

**Supplementary Figure 10:** The extracted ion chromatogram (EIC) of 11 sterols in retention time quality control (RTQC) sample.

**Supplementary Figure 11:** The information on calibration sample plots, fitting line, equations and R2 values of 21 calibration curves in mouse cerebral nuclei region samples.

## List of Supplementary Tables

**Supplementary Table 1**: The information on LM_ID, name, m/z and RT of 11 sterol lipids in the RTQC sample.

**Supplementary Table 2**: The information on LM_ID, name, equation, $R^2$ value, dynamic range and LOD of 21 calibration curves in mouse cerebral nuclei region samples.

**Supplementary Table 3:** The LM_ID of quantified sterols with level 2 identifications and their reference sterols with calibration curves.

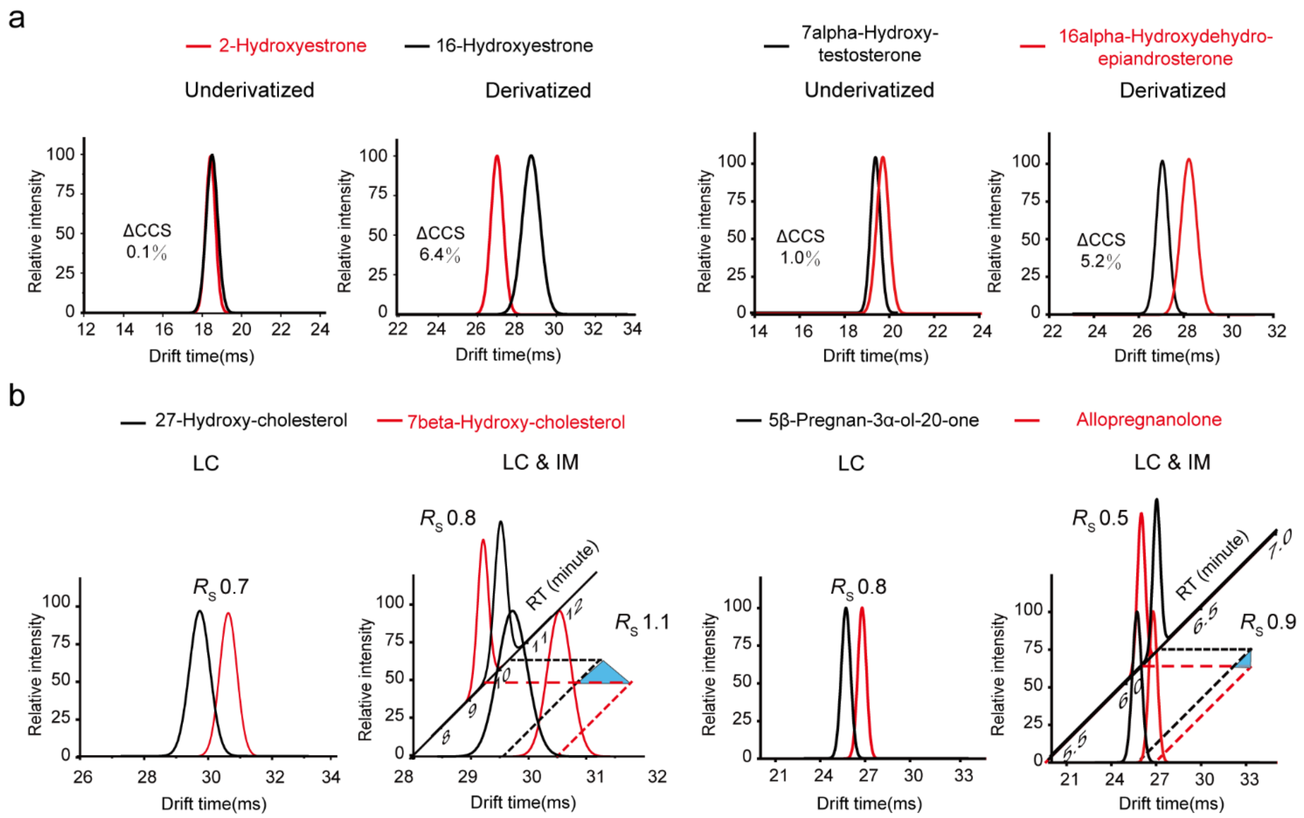


**Supplementary Figure 1.**

**Examples for improved separation of sterol isomers with derivatization and IM-MS.** (**a**) Overlay of IM mobiligrams of underivatized and derivatized sterol isomers of 2-hydroxyestrone and 16-hydroxyestrone, 7alpha-hydroxy-testosterone and 16alpha-hydroxydehydro-epiandrosterone. (**b**) The peak resolutions ($R_S$) in LC separation, IM separation and LC-IM based two-dimensional separation for sterol isomers of 27-hydroxy-cholesterol and 7beta-hydroxy-cholesterol, 5β-pregnan-3α-ol-20-one and allopregnanolone after derivatization. Source data are provided as a Source Data file.

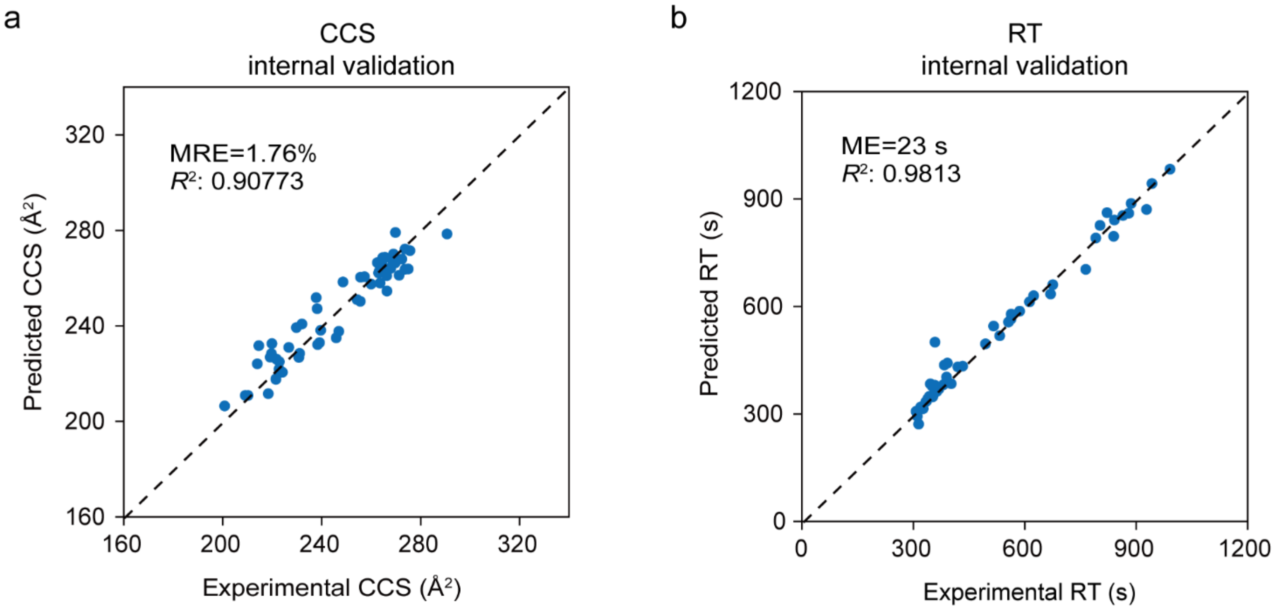


**Supplementary Figure 2.**

**The internal validations of CCS prediction and retention time prediction using the training data set.** (**a**) The internal validation of machine-learning based prediction of CCS values of sterols (n=57). (**b**) The internal validation of machine-learning based prediction of retention times of sterols (n=57). Source data are provided as a Source Data file.

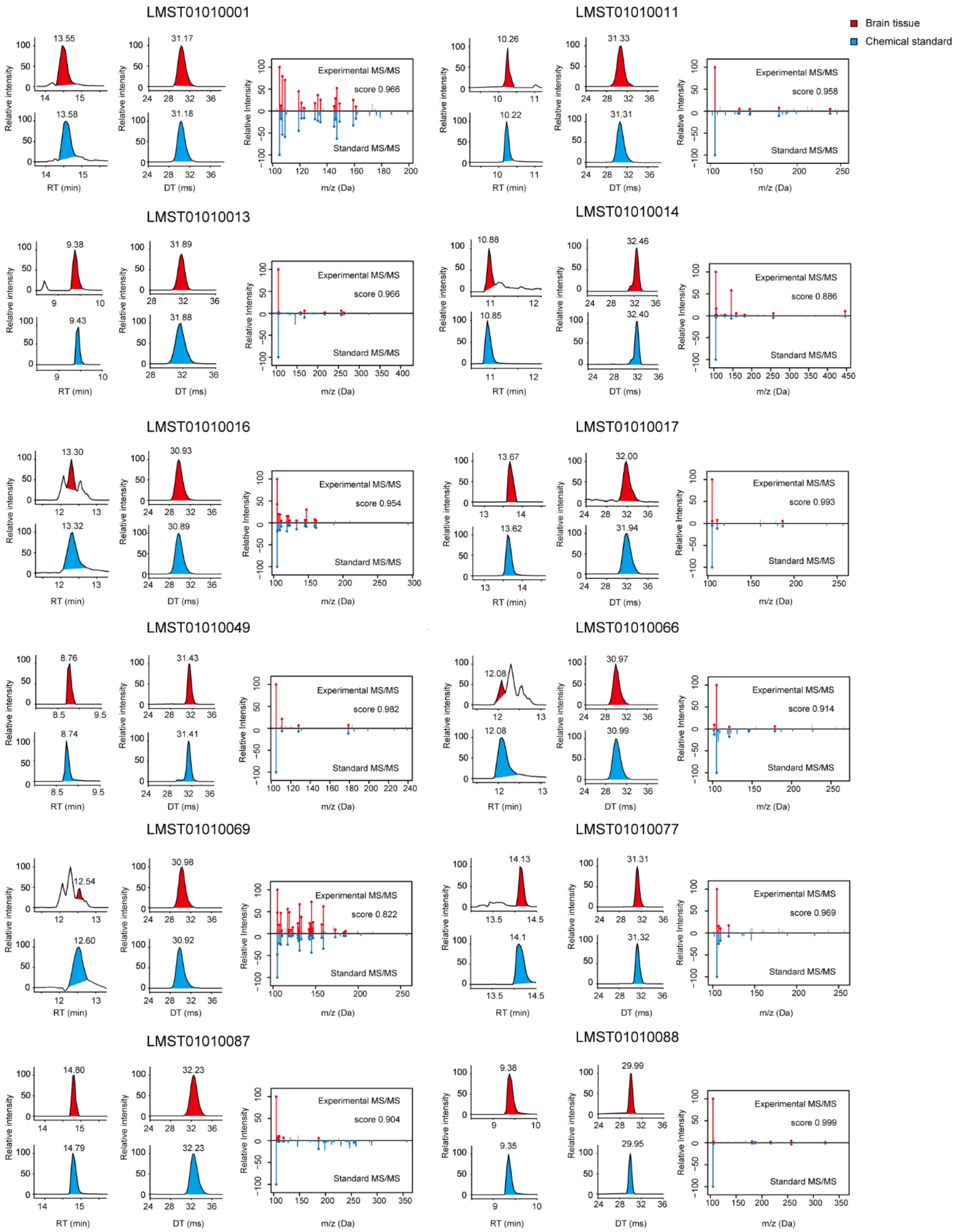


**Supplementary Figure 3.**

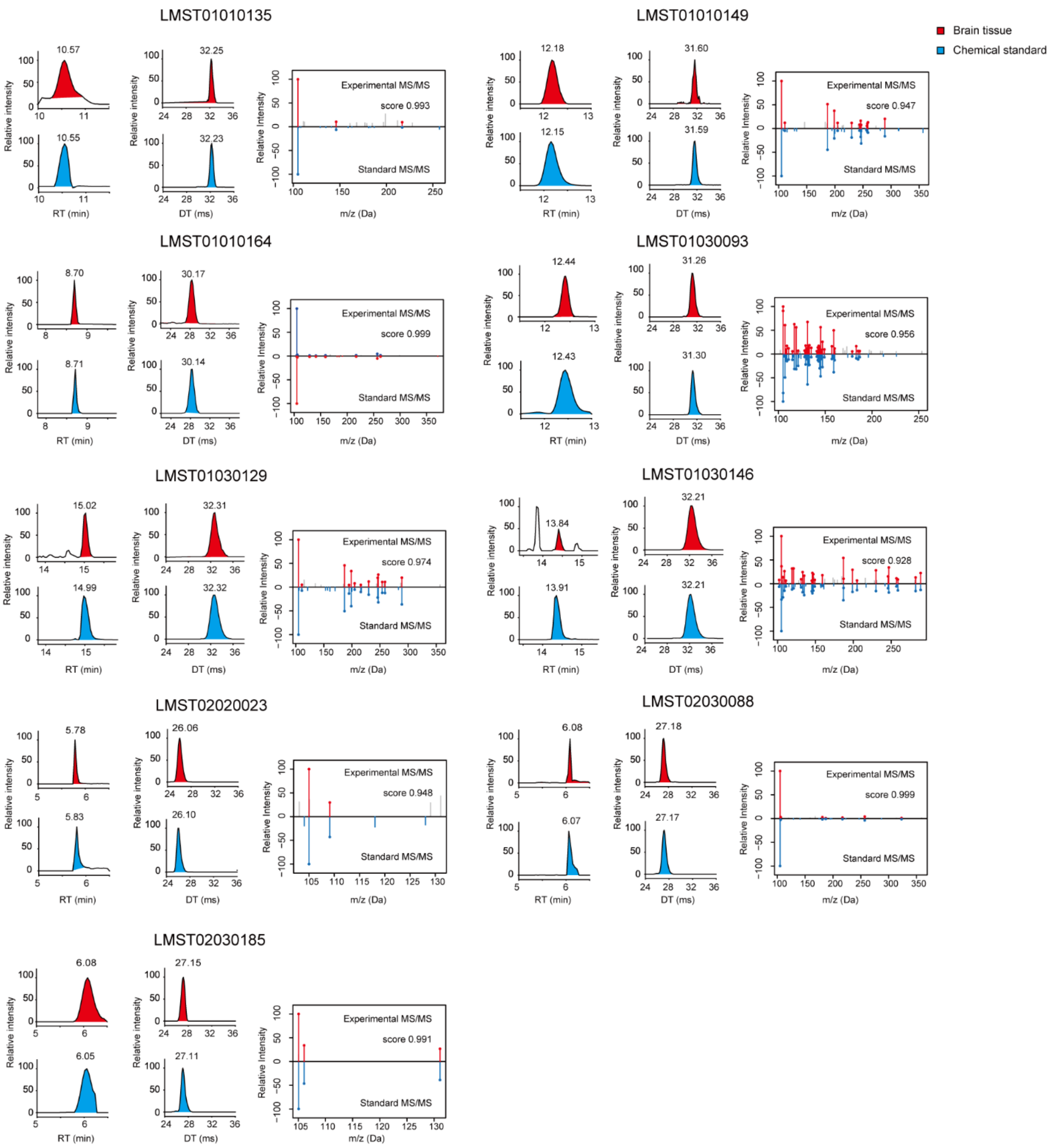


**Supplementary Figure 3 (continued).**

**Validations of 21 sterol lipids in brain tissues using purchased chemical standards.** The RT alignment, DT alignment and mirror plot of MS/MS spectra of 21 sterol lipids in brain tissues in brain sample and chemical standards. Source data are provided as a Source Data file.

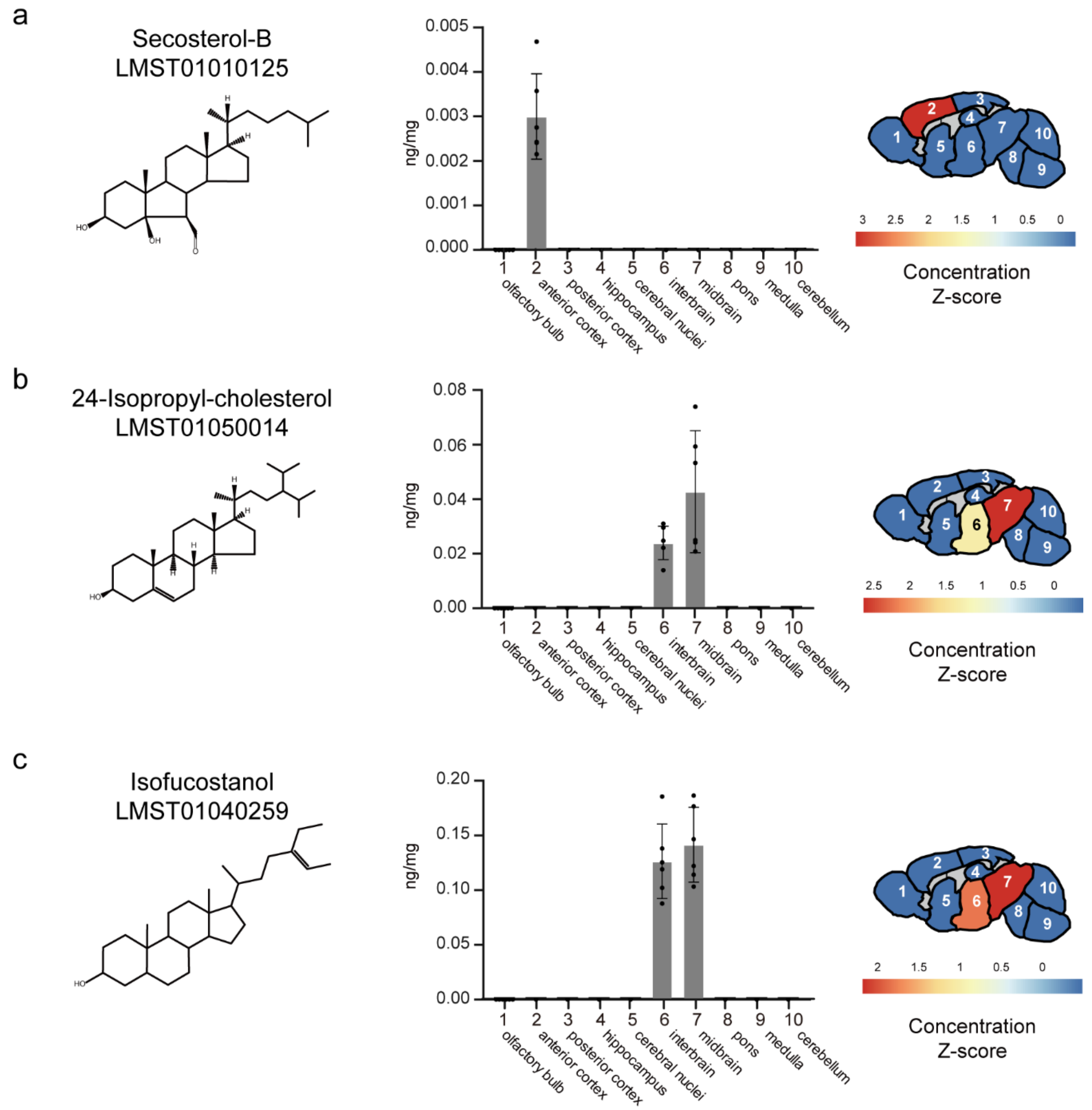


**Supplementary Figure 4.**

**Concentrations of secosterol-B, 24-isopropyl-cholesterol, and isofucostanol measured in brain regions.** (**a**) The structures and concentrations of secosterol-B measured in ten brain regions. (**b**) The structures and concentrations of 24-isopropyl-cholesterol measured in ten brain regions. (**c**) The structures and concentrations of isofucostanol measured in ten brain regions. Data are presented as mean values +/- SD (n=6 biologically independent samples for each group). Source data are provided as a Source Data file.

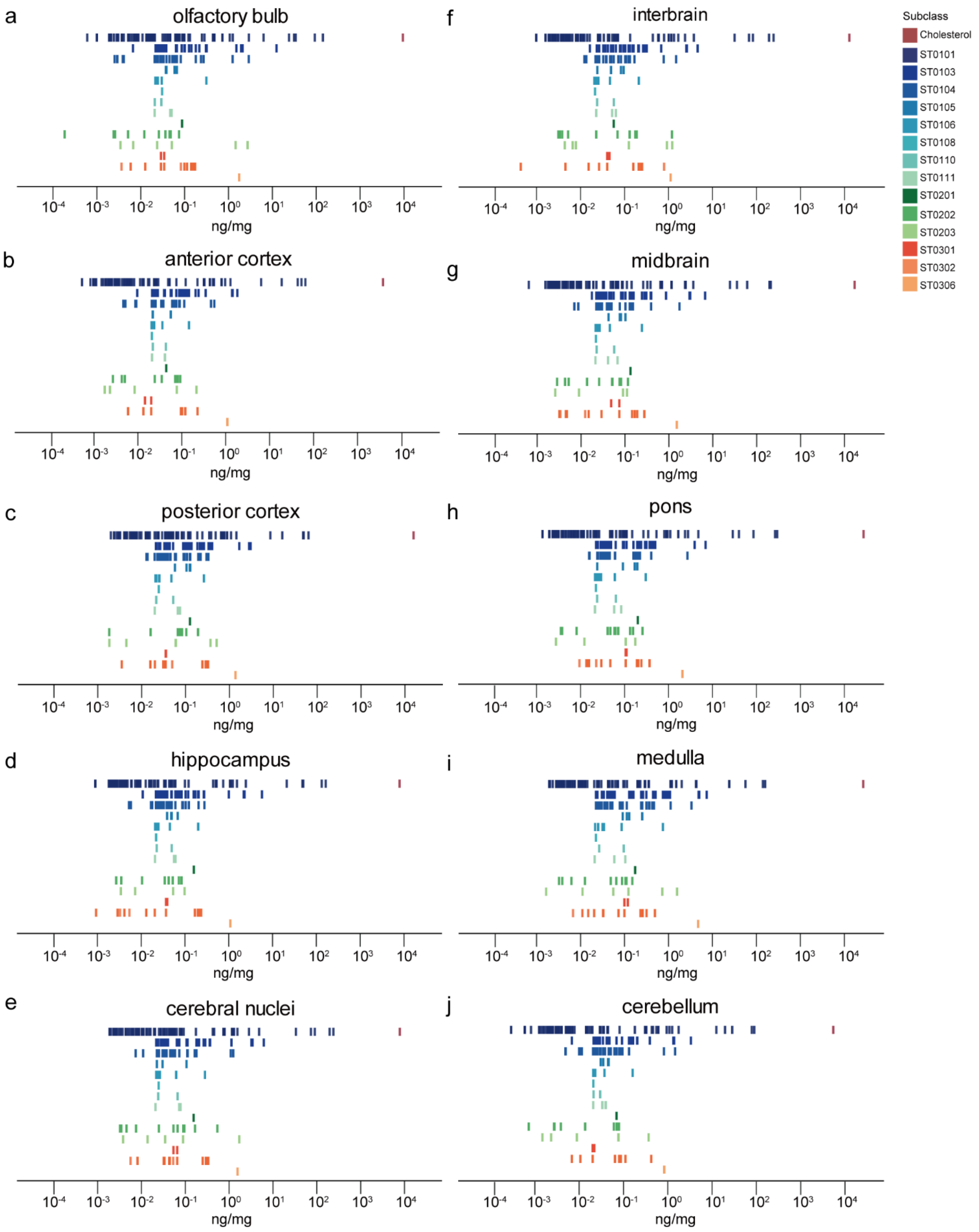


**Supplementary Figure 5.**

**Concentration ranges of sterol lipids measured in ten brain regions.** (**a**) olfactory bulb. (**b**) anterior cortex. (**c**) posterior cortex. (**d**) hippocampus. (**e**) cerebral nuclei. (**f**) interbrain. (**g**) midbrain. (**h**) pons. (**i**) medulla. (**j**) cerebellum. Source data are provided as a Source Data file.

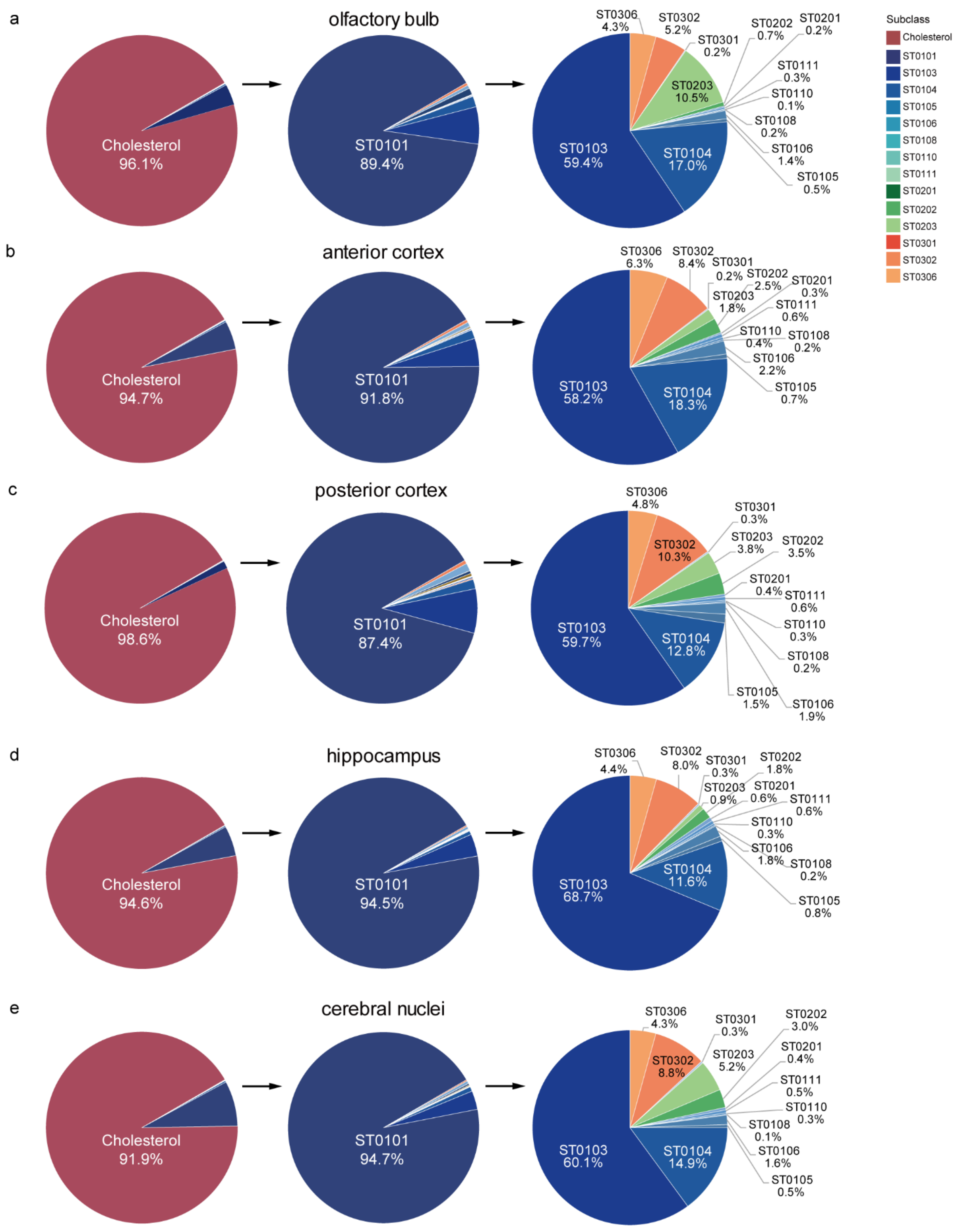


**Supplementary Figure 6.**

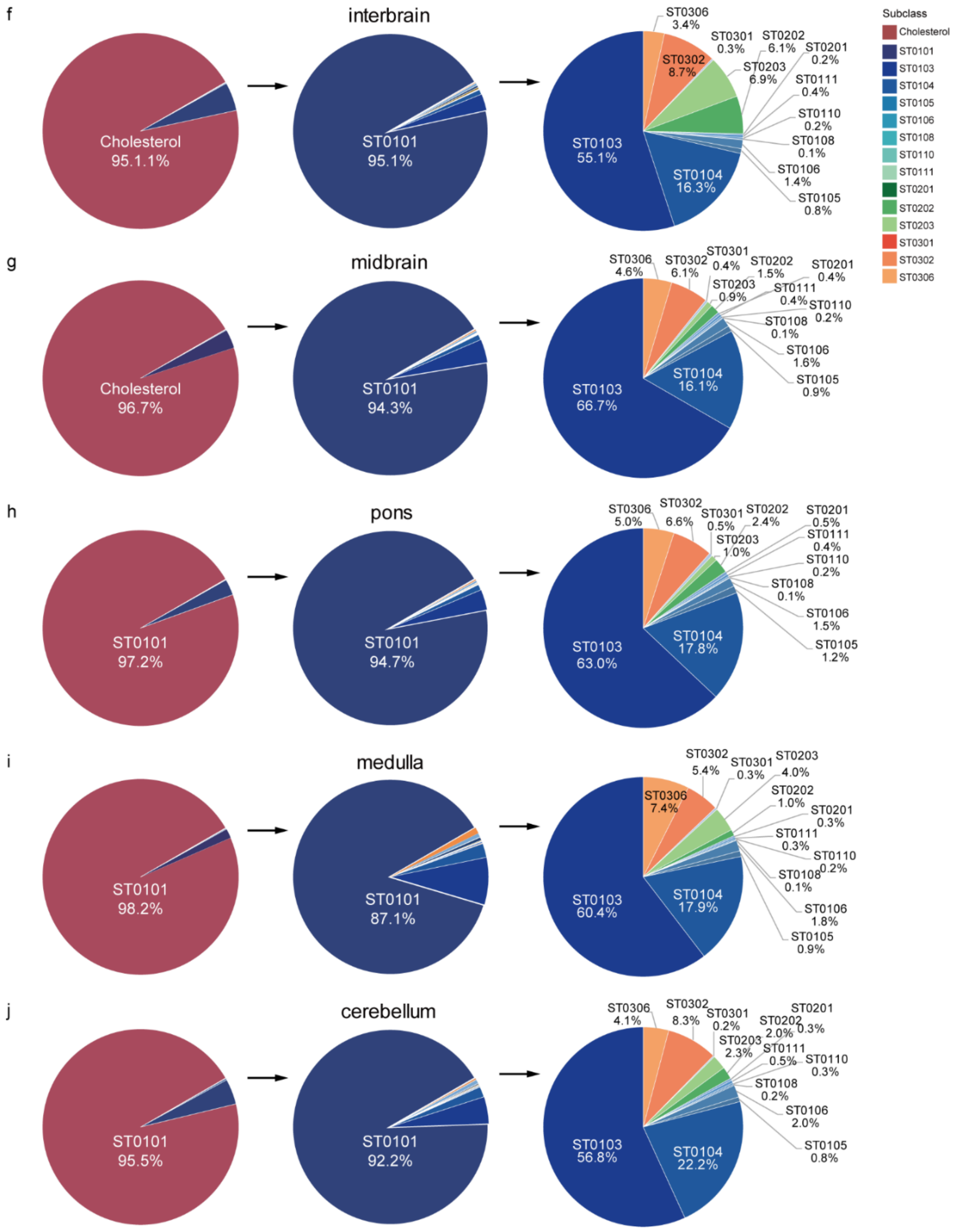


**Supplementary Figure 6 (continued).**

**Percentage compositions of different sterol subclasses measured in ten brain regions.** (**a**) olfactory bulb. (**b**) anterior cortex. (**c**) posterior cortex. (**d**) hippocampus. (**e**) cerebral nuclei. (**f**) interbrain. (**g**) midbrain. (**h**) pons. (**i**) medulla. (**j**) cerebellum. Source data are provided as a Source Data file.

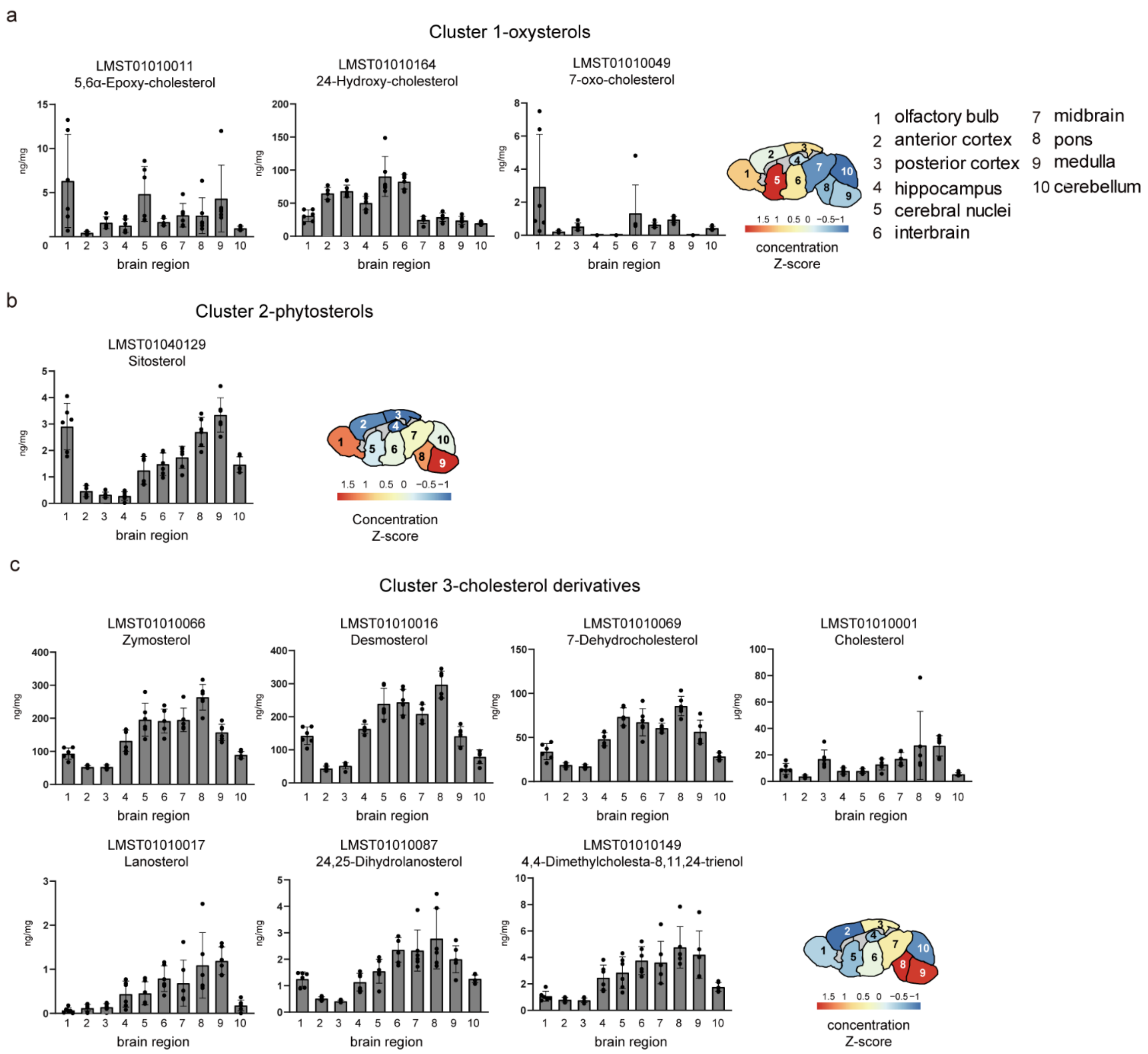


**Supplementary Figure 7.**

**Concentration distributions of oxysterols, phytosterols and cholesterol derivatives in mouse brain regions.** (**a**) The bar plot and concentration distributions of 3 oxysterols measured in ten brain regions. (**b**) The bar plot and concentration distribution of 1 phytosterol (LMST01040129) measured in ten brain regions. (**c**) The bar plot and concentration distributions of cholesterol and 6 cholesterol derivatives measured in ten brain regions. Data are presented as mean values +/- SD (n=6 biologically independent samples for each group). Source data are provided as a Source Data file.

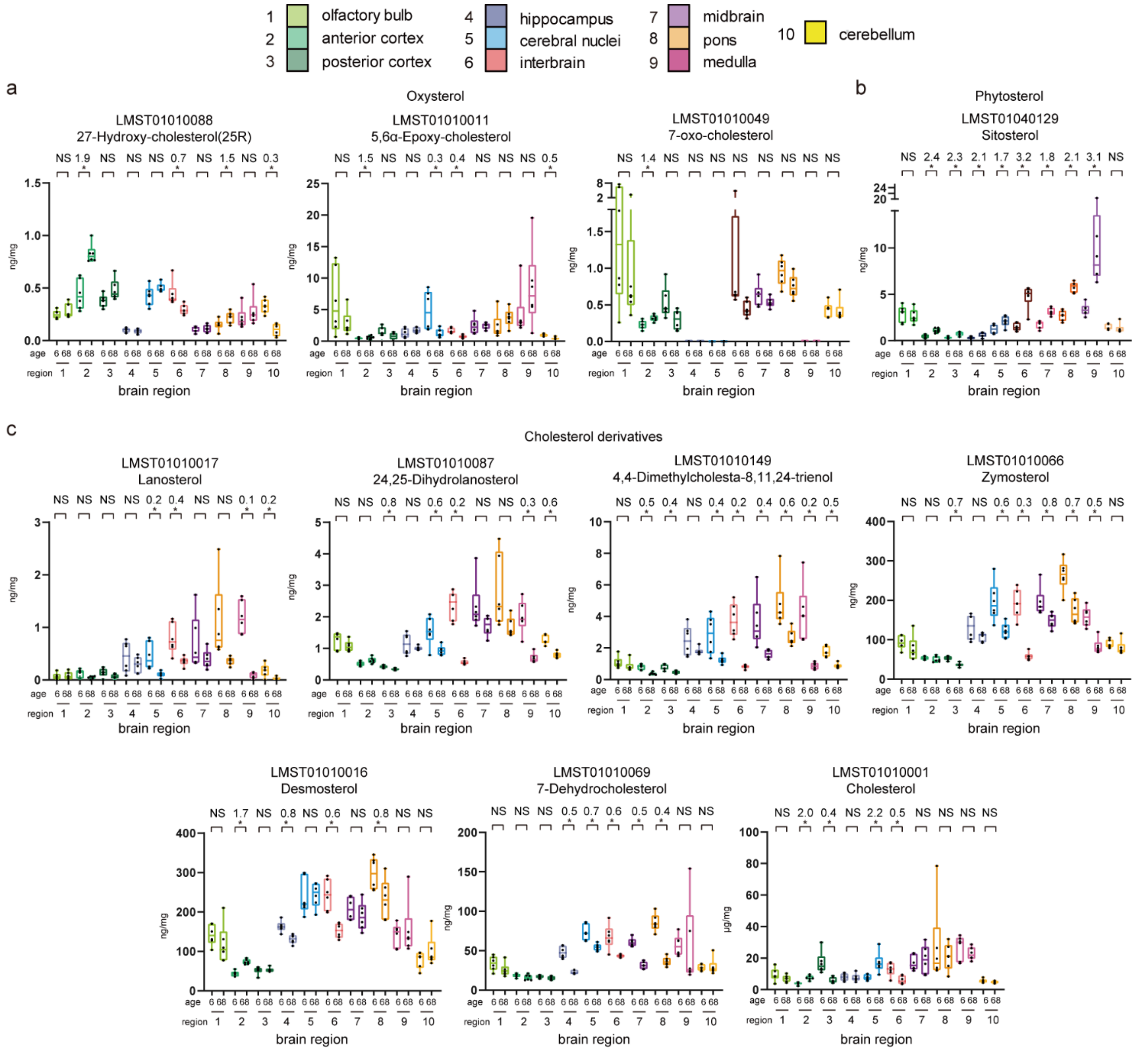


**Supplementary Figure 8.**

**Amplitude changes of oxysterols, phytosterols and cholesterol derivatives measured in mouse brain regions.** (**a**) Concentrations and fold-changes of 3 oxysterols in the brain regions of young and old mice (n=6 biologically independent samples for each group, two-sided Student's *t*-test; *p*-value adjusted using FDR; *p*-value < 0.05: *; *p*-value ≥ 0.05: NS). (**b**) Concentrations and fold-changes of 1 phytosterols in the brain regions of young and old mice (n=6 biologically independent samples for each group, two-sided Student's *t*-test; *p*-value adjusted using FDR; *p*-value < 0.05: *; *p*-value ≥ 0.05: NS). (**c**) Concentrations and fold-changes of cholesterol and 6 cholesterol derivatives in the brain regions of young and old mice (n=6 biologically independent samples for each group, two-tailed Student's t-test; p value adjusted using FDR; *p*-value < 0.05: *; p value ≥ 0.05: NS). The lower, middle, and upper lines in box plots **(a-c)** correspond to 25th, 50th, and 75th quartiles, and the whiskers extend to the most extreme data point within 1.5 interquartile range (IQR). Source data are provided as a Source Data file.

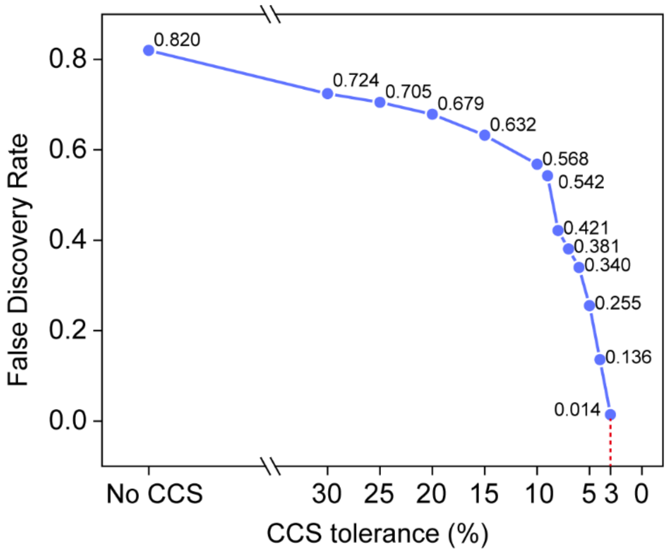


**Supplementary Figure 9.**

**The estimation of false discovery rates (FDR) under different CCS matching tolerances using the extended ST library in mouse olfactory bulb region samples.** The estimation of false discovery rates (FDR) under different CCS matching tolerances (No, 30%, 25%, 20%, 15%, 10%, 9%, 8%, 7%, 6%, 5%, 4%, 3%). Source data are provided as a Source Data file.

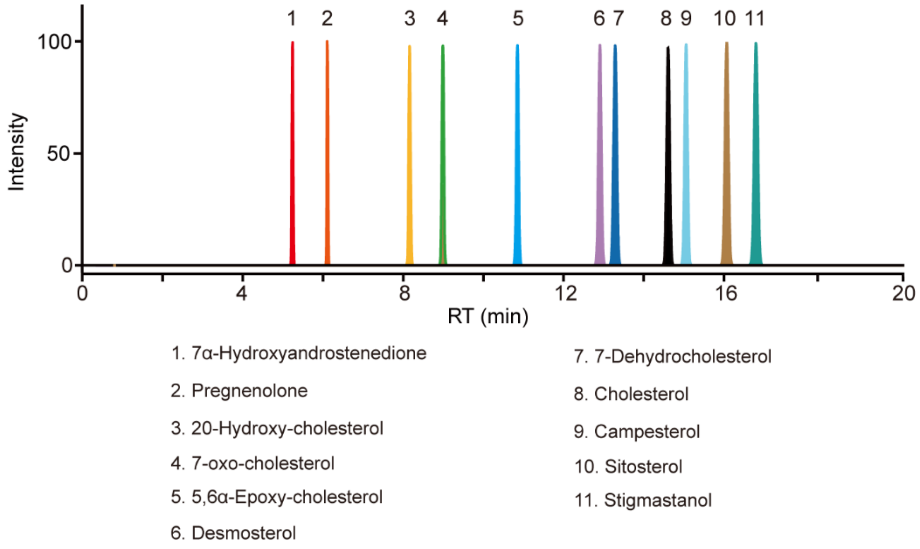


**Supplementary Figure 10.**

**The extracted ion chromatogram (EIC) of 11 sterols in retention time quality control (RTQC) sample.** Source data are provided as a Source Data file.

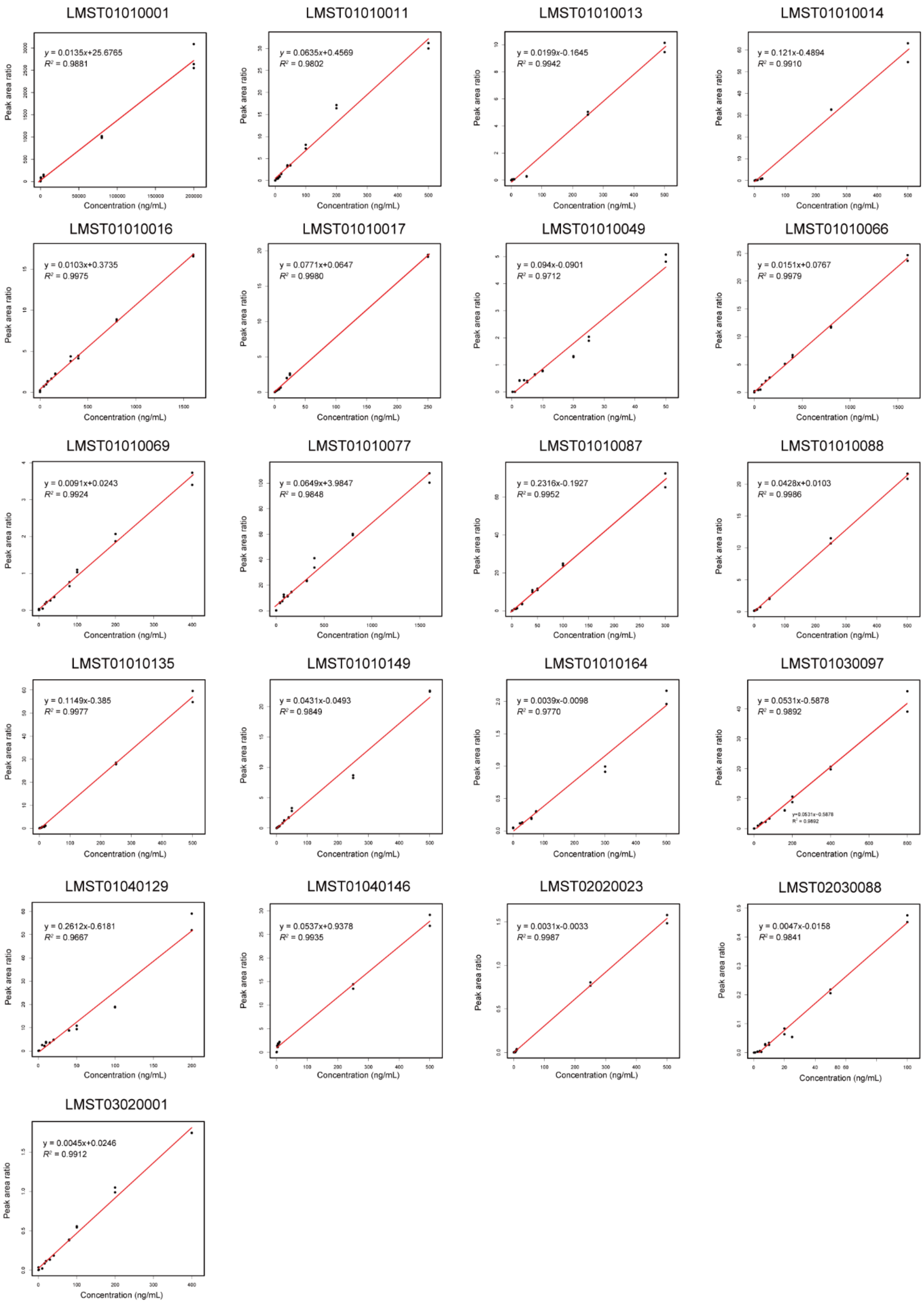


**Supplementary Figure 11.**

**The information on calibration sample plots, fitting line, equations and *$R^2$* values of 21 calibration curves in mouse cerebral nuclei region samples.** The x axes are concentration (ng/mg) and the y axes are peak area ratio (sterol/IS). Scatter plots indicate calibration samples. The red lines indicate the linear fitting. The equations and $R^2$ values are generated by linear regression fitting. Source data are provided as a Source

Data file.

**Supplementary Table 1.**

**The information on LM_ID, name, m/z and RT of 11 sterol lipids in the RTQC sample.** Source data are provided as a Source Data file.

| LM_ID | Sterol lipid name | Accurate *m/z* after derivatization | RT (min) |
|---|---|---|---|
| LMST02020071 | 7α-Hydroxyandrostenedione | 430.1994 | 5.27 |
| LMST02030088 | Pregnenolone | 444.2514 | 6.14 |
| LMST01010201 | 20-Hydroxy-cholesterol | 530.3610 | 8.24 |
| LMST01010049 | 7-oxo-cholesterol | 528.3453 | 9.19 |
| LMST01010011 | 5,6α-Epoxy-cholesterol | 530.3610 | 10.92 |
| LMST01010016 | Desmosterol | 512.3504 | 13.06 |
| LMST01010069 | 7-Dehydrocholesterol | 512.3504 | 13.35 |
| LMST01010001 | Cholesterol | 514.3661 | 14.34 |
| LMST01030097 | Campesterol | 528.3817 | 15.11 |
| LMST01040129 | Sitosterol | 542.3973 | 16.01 |
| LMST01040266 | Stigmastanol | 544.4132 | 16.87 |

Note: The table provided the LM_IDs, sterol lipid names, accurate m/z values after derivatization and RT of 11 sterol lipids in the RTQC sample.

**Supplementary Table 2.**

**The information on LM_ID, name, equation, $R^2$ value, dynamic range and LOD of 21 calibration curves in mouse cerebral nuclei region samples.** Source data are provided as a Source Data file.

| LM_ID | Sterol lipid name | Equation | $R^2$ | Dynamic range (ng/mL) | LOD (ng/mL) |
|---|---|---|---|---|---|
| LMST01010001 | Cholesterol | y=0.0135x+25.6765 | 0.9881 | 10-40000 | 10 |
| LMST01010011 | 5,6alpha-Epoxy-cholesterol | y=0.0635x+0.4569 | 0.9802 | 0.25-500 | 0.25 |
| LMST01010013 | 7alpha-Hydroxy-cholesterol | y=0.0199x-0.1645 | 0.9942 | 0.25-500 | 0.25 |
| LMST01010014 | 4beta-Hydroxy-cholesterol | y=0.121x-0.4894 | 0.9910 | 0.25-500 | 0.25 |
| LMST01010016 | Desmosterol | y=0.0103x+0.3735 | 0.9975 | 0.25-500 | 0.25 |
| LMST01010017 | Lanosterol | y=0.0771x+0.0647 | 0.9980 | 0.25-250 | 0.25 |
| LMST01010049 | 7-oxo-cholesterol | y=0.094x-0.0901 | 0.9712 | 0.25-50 | 0.25 |
| LMST01010066 | Zymosterol | y=0.0151x+0.0767 | 0.9979 | 0.25-1600 | 0.25 |
| LMST01010069 | 7-Dehydrocholesterol | y=0.0091x+0.0243 | 0.9924 | 0.25-400 | 0.25 |
| LMST01010077 | Dihydrocholesterol | y=0.0649x+3.9847 | 0.9848 | 0.25-1600 | 0.25 |
| LMST01010087 | 24,25-Dihydrolanosterol | y=0.2316x-0.1927 | 0.9952 | 0.25-300 | 0.25 |
| LMST01010088 | 27-Hydroxy-cholesterol | y=0.0428x+0.0103 | 0.9986 | 0.25-500 | 0.25 |
| LMST01010135 | 6alpha-Hydroxy-cholestanol | y=0.1149x-0.385 | 0.9977 | 0.25-500 | 0.25 |
| LMST01010149 | 4,4-Dimethylcholesta-8,11,24-trienol | y=0.0431x-0.0493 | 0.9849 | 0.25-500 | 0.25 |
| LMST01010164 | 24-Hydroxy-cholesterol | y=0.0039x-0.0098 | 0.9770 | 0.25-500 | 0.25 |
| LMST01030097 | Campesterol | y=0.0531x-0.5878 | 0.9892 | 0.25-800 | 0.25 |
| LMST01040129 | Sitosterol | y=0.2612x-0.6181 | 0.9667 | 0.25-300 | 0.25 |
| LMST01040146 | Fucosterol | y=0.0537x+0.9378 | 0.9935 | 0.25-500 | 0.25 |
| LMST02020023 | Epiandrosterone | y=0.0031x-0.0033 | 0.9987 | 0.25-500 | 0.25 |
| LMST02030088 | Pregnenolone | y=0.0047x-0.0158 | 0.9841 | 0.25-100 | 0.25 |
| LMST03020001 | Vitamin D3 | y=0.0045x+0.0246 | 0.9912 | 0.25-400 | 0.25 |

Note: The table also show the equations and $R^2$ values that generated by linear regression fitting in calibration samples of mouse cerebral nuclei region. The limit of detection (LOD) of each calibration curves in mouse cerebral nuclei region are provided in table.

**Supplementary Table 3.**

**The LM_ID of quantified sterols with level 2 identifications and their reference sterols with calibration curves.** Source data are provided as a Source Data file.

| Sterols with level 2 identification (LM_ID) | Reference sterols with calibrations curves (LM_ID) |
|---|---|
| LMST01010010 | LMST01010011 |
| LMST01010012 | LMST01010011 |
| LMST01010077 | LMST01010087 |
| LMST01010086 | LMST01010088 |
| LMST01010094 | LMST01010087 |
| LMST01010095 | LMST01010087 |
| LMST01010099 | LMST01010087 |
| LMST01010121 | LMST01010087 |
| LMST01010125 | LMST01010087 |
| LMST01010127 | LMST01010087 |
| LMST01010133 | LMST01010049 |
| LMST01010139 | LMST01010087 |
| LMST01010140 | LMST01010087 |
| LMST01010169 | LMST01010087 |
| LMST01010171 | LMST01010087 |
| LMST01010176 | LMST01010087 |
| LMST01010201 | LMST01010049 |
| LMST01010202 | LMST01010087 |
| LMST01010207 | LMST01010087 |
| LMST01010224 | LMST01010087 |
| LMST01010225 | LMST01010087 |
| LMST01010226 | LMST01010087 |
| LMST01010234 | LMST01010087 |
| LMST01010254 | LMST01010087 |
| LMST01010270 | LMST01010049 |
| LMST01010271 | LMST01010049 |
| LMST01010274 | LMST01010270 |
| LMST01010275 | LMST01010088 |
| LMST01010276 | LMST01010049 |
| LMST01010279 | LMST01010088 |
| LMST01010280 | LMST01010088 |
| LMST01010282 | LMST01010087 |
| LMST01010284 | LMST01010087 |
| LMST01010286 | LMST01010087 |
| LMST01010298 | LMST01010049 |
| LMST01010300 | LMST01010049 |
| LMST01010306 | LMST01010087 |
| LMST01010307 | LMST01010049 |
| LMST01010308 | LMST01010088 |

| | |
|---|---|
| LMST01010310 | LMST01010049 |
| LMST01010315 | LMST01010049 |
| LMST01010316 | LMST01010087 |
| LMST01010330 | LMST01010087 |
| LMST01010333 | LMST01010087 |
| LMST01010345 | LMST01010270 |
| LMST01010351 | LMST01010087 |
| LMST01010354 | LMST01010270 |
| LMST01010355 | LMST01010270 |
| LMST01010356 | LMST01010270 |
| LMST01010357 | LMST01010088 |
| LMST01010358 | LMST01010088 |
| LMST01010359 | LMST01010088 |
| LMST01010386 | LMST01010088 |
| LMST01010387 | LMST01010049 |
| LMST01030094 | LMST01030097 |
| LMST01030095 | LMST01030097 |
| LMST01030096 | LMST01030097 |
| LMST01030098 | LMST01030097 |
| LMST01030101 | LMST01030097 |
| LMST01030102 | LMST01030097 |
| LMST01030103 | LMST01030097 |
| LMST01030105 | LMST01030097 |
| LMST01030107 | LMST01030097 |
| LMST01030109 | LMST01030097 |
| LMST01030110 | LMST01030097 |
| LMST01030115 | LMST01030097 |
| LMST01030117 | LMST01030097 |
| LMST01030119 | LMST01030097 |
| LMST01030135 | LMST01030097 |
| LMST01030137 | LMST01030097 |
| LMST01030142 | LMST01030097 |
| LMST01030145 | LMST01030097 |
| LMST01030147 | LMST01030097 |
| LMST01031021 | LMST01030097 |
| LMST01031024 | LMST01030097 |
| LMST01031027 | LMST01030097 |
| LMST01031033 | LMST01030097 |
| LMST01031035 | LMST01030097 |
| LMST01031037 | LMST01030097 |
| LMST01031038 | LMST01030097 |
| LMST01031039 | LMST01030097 |
| LMST01031043 | LMST01030097 |
| LMST01031044 | LMST01030097 |

| | |
|---|---|
| LMST01031046 | LMST01030097 |
| LMST01031049 | LMST01030097 |
| LMST01031055 | LMST01030097 |
| LMST01031056 | LMST01030097 |
| LMST01031058 | LMST01030097 |
| LMST01031066 | LMST01030097 |
| LMST01031110 | LMST01030097 |
| LMST01040121 | LMST01030097 |
| LMST01040122 | LMST01030097 |
| LMST01040123 | LMST01030097 |
| LMST01040124 | LMST01030097 |
| LMST01040125 | LMST01030097 |
| LMST01040126 | LMST01030097 |
| LMST01040127 | LMST01030097 |
| LMST01040128 | LMST01030097 |
| LMST01040130 | LMST01030097 |
| LMST01040133 | LMST01030097 |
| LMST01040134 | LMST01030097 |
| LMST01040135 | LMST01030097 |
| LMST01040137 | LMST01030097 |
| LMST01040139 | LMST01030097 |
| LMST01040140 | LMST01030097 |
| LMST01040141 | LMST01030097 |
| LMST01040148 | LMST01030097 |
| LMST01040151 | LMST01030097 |
| LMST01040156 | LMST01030097 |
| LMST01040157 | LMST01030097 |
| LMST01040161 | LMST01030097 |
| LMST01040165 | LMST01030097 |
| LMST01040168 | LMST01030097 |
| LMST01040174 | LMST01030097 |
| LMST01040189 | LMST01030097 |
| LMST01040196 | LMST01030097 |
| LMST01040238 | LMST01030097 |
| LMST01040239 | LMST01030097 |
| LMST01040242 | LMST01030097 |
| LMST01040248 | LMST01030097 |
| LMST01040250 | LMST01030097 |
| LMST01040257 | LMST01030097 |
| LMST01040258 | LMST01030097 |
| LMST01040259 | LMST01030097 |
| LMST01050014 | LMST01030097 |
| LMST01050015 | LMST01030097 |
| LMST01060001 | LMST01030097 |

| | |
|---|---|
| LMST01060005 | LMST01030097 |
| LMST01060009 | LMST01030097 |
| LMST01060010 | LMST01030097 |
| LMST01060013 | LMST01030097 |
| LMST01080007 | LMST01030097 |
| LMST01080019 | LMST01030097 |
| LMST01100008 | LMST01030097 |
| LMST01110006 | LMST01030097 |
| LMST01110008 | LMST01030097 |
| LMST01110010 | LMST01030097 |
| LMST02010036 | LMST02020023 |
| LMST02020004 | LMST02020023 |
| LMST02020023 | LMST02020023 |
| LMST02020028 | LMST02020023 |
| LMST02020044 | LMST02020023 |
| LMST02020086 | LMST02020023 |
| LMST02020097 | LMST02020023 |
| LMST02020098 | LMST02020023 |
| LMST02020099 | LMST02020023 |
| LMST02020102 | LMST02020023 |
| LMST02020105 | LMST02020023 |
| LMST02020107 | LMST02020023 |
| LMST02020111 | LMST02020023 |
| LMST02020112 | LMST02020023 |
| LMST02020114 | LMST02020023 |
| LMST02030088 | LMST02030088 |
| LMST02030134 | LMST02020023 |
| LMST02030152 | LMST02020023 |
| LMST02030153 | LMST02020023 |
| LMST03010056 | LMST03020001 |
| LMST03020004 | LMST03020001 |
| LMST03020009 | LMST03020001 |
| LMST03020010 | LMST03020001 |
| LMST03020015 | LMST03020001 |
| LMST03020016 | LMST03020001 |
| LMST03020017 | LMST03020001 |
| LMST03020044 | LMST03020001 |
| LMST03020140 | LMST03020001 |
| LMST03020197 | LMST03020001 |
| LMST03020202 | LMST03020001 |
| LMST03020227 | LMST03020001 |
| LMST03020228 | LMST03020001 |
| LMST03020229 | LMST03020001 |
| LMST03020230 | LMST03020001 |

| | |
|---|---|
| LMST03020252 | LMST03020001 |
| LMST03020253 | LMST03020001 |
| LMST03020334 | LMST03020001 |
| LMST03020335 | LMST03020001 |
| LMST03020663 | LMST03020001 |
| LMST03060002 | LMST03020001 |

Note: The concentrations of sterol lipids with level 2 identifications in brain samples were interpolated from the calibration curves of sterol lipid standards within the same sterol subclass
