# Supplementary material for: Ion mobility-based sterolomics reveals spatially and temporally distinctive sterol lipids in the mouse brain
Source: Nat Commun. 2021 Jul 15;12:4343. doi: 10.1038/s41467-021-24672-x (PMC8282640; doi:10.1038/s41467-021-24672-x)
Supplement: Supplementary file 16 — Reporting Summary [file 41467_2021_24672_MOESM16_ESM.pdf]

nature portfolio

Corresponding author(s): Dr. Zheng-Jiang Zhu

Last updated by author(s): Jun 28, 2021

Reporting Summary

Nature Portfolio wishes to improve the reproducibility of the work that we publish. This form provides structure for consistency and transparency in reporting. For further information on Nature Portfolio policies, see our Editorial Policies and the Editorial Policy Checklist.

Statistics

For all statistical analyses, confirm that the following items are present in the figure legend, table legend, main text, or Methods section.

n/a	Confirmed	
☐	☒	The exact sample size (*n*) for each experimental group/condition, given as a discrete number and unit of measurement
☐	☒	A statement on whether measurements were taken from distinct samples or whether the same sample was measured repeatedly
☐	☒	The statistical test(s) used AND whether they are one- or two-sided<br>*Only common tests should be described solely by name; describe more complex techniques in the Methods section.*
☒	☐	A description of all covariates tested
☐	☒	A description of any assumptions or corrections, such as tests of normality and adjustment for multiple comparisons
☐	☒	A full description of the statistical parameters including central tendency (e.g. means) or other basic estimates (e.g. regression coefficient) AND variation (e.g. standard deviation) or associated estimates of uncertainty (e.g. confidence intervals)
☐	☒	For null hypothesis testing, the test statistic (e.g. *F*, *t*, *r*) with confidence intervals, effect sizes, degrees of freedom and *P* value noted<br>*Give P values as exact values whenever suitable.*
☒	☐	For Bayesian analysis, information on the choice of priors and Markov chain Monte Carlo settings
☒	☐	For hierarchical and complex designs, identification of the appropriate level for tests and full reporting of outcomes
☐	☒	Estimates of effect sizes (e.g. Cohen's *d*, Pearson's *r*), indicating how they were calculated

Our web collection on statistics for biologists contains articles on many of the points above.

Software and code

Policy information about availability of computer code

Data collection	These tools were used in data collection: MassHunter Workstation Data Acquisition Software (Version B.08.00, Agilent Technologies, USA); LIPID MAPS Structure Database[https://www.lipidmaps.org/data/structure/download.php] (accessed on December 16th, 2016); PubChem Database[https://pubchem.ncbi.nlm.nih.gov/] (accessed on December 26th, 2016); R package “rcdk” (version 3.3.8); Lasso and Elastic-Net Regularized Generalized Linear Models, R package “glmnet” (version 4.1)
Data analysis	IM-MS Reprocessor (Version B.08.00, Agilent Technologies); PNNL PreProcessor (Version 2018.06.02); IM-MS Browser software (Version B.08.00, Agilent Technologies); Mass Profiler (Version B.08.01, Agilent Technologies); Cytoscape (version 3.7.2); The source code of Sterol4DAnalyzer was provided in GitHub [https://github.com/ZhuMetLab/Sterol4DAnalyzer] with a DOI number [https://doi.org/10.5281/zenodo.4743402].

For manuscripts utilizing custom algorithms or software that are central to the research but not yet described in published literature, software must be made available to editors and reviewers. We strongly encourage code deposition in a community repository (e.g. GitHub). See the Nature Portfolio guidelines for submitting code & software for further information.

Data

Policy information about availability of data

All manuscripts must include a data availability statement. This statement should provide the following information, where applicable:

- Accession codes, unique identifiers, or web links for publicly available datasets
- A description of any restrictions on data availability
- For clinical datasets or third party data, please ensure that the statement adheres to our policy

The raw data files generated in this study have been deposited in the National Omics Data Encyclopedia under accession code OEP002113 [https://

www.biosino.org/node/project/detail/OEP002113]. The converted data (MS1 peak table and MS/MS spectra files) generated in this study have been deposited in the MetaboLights under accession code MTBLS2457 [https://www.ebi.ac.uk/metabolights/MTBLS2457/descriptors]. The information on 97 sterol lipids in the standard ST library and 2,068 sterol lipids in the extended ST library generated in this study is provided in the Supplementary Data 3. The sterol identification and quantification results generated in this study are provided in the Supplementary Data 6-8. The interactive mouse brain atlas of sterol lipids is provided in the Brain Sterol Atlas website [http://mousebrainatlas.zhulab.cn/]. The simplified molecular input line entry specification (SMILES) of 97 sterol lipids were collected from LIPID MAPS Structure Database [https://www.lipidmaps.org/data/structure/download.php] (accessed on December 16th, 2016) and PubChem [https://pubchem.ncbi.nlm.nih.gov/] (accessed on December 26th, 2016) to describe the chemical structures. Source data are provided with this paper.

Field-specific reporting

Please select the one below that is the best fit for your research. If you are not sure, read the appropriate sections before making your selection.

☒ Life sciences ☐ Behavioural & social sciences ☐ Ecological, evolutionary & environmental sciences

For a reference copy of the document with all sections, see nature.com/documents/nr-reporting-summary-flat.pdf

Life sciences study design

All studies must disclose on these points even when the disclosure is negative.

Sample size	The experiments of aging mouse described were performed with six biologically independent samples for each group. The experiments of human plasma described were performed with three technical replicates. No sample-size calculation was performed here. The sample sizes were chosen because they are the standard number for most biological studies in metabolomics and lipidomics.
Data exclusions	No samples were excluded from analysis in this study.
Replication	The human plasma samples (n=3 technically replicated samples) were used for data analysis in Figure 4. The mouse brain tissues (n=6 biologically independent samples) and mouse liver tissue (n=6 biologically independent samples) were used for data analysis in Figure 4. Each sample was analyzed once by LC-IM-MS. The mouse brain tissues (6 and 68 weeks old; n=6 biologically independent samples for each group) were used for data analysis in Figures 5 and 6. Each sample was analyzed once by LC-IM-MS.
Randomization	For aging mouse brain datasets, samples were assigned randomly to acquire LC-IM-MS data. For other datasets, the randomization is not required, because only one standard sample was used.
Blinding	At the time of sample acquisition and processing, scientists were completely unaware of the sample group. The data analyses were blinded.

Reporting for specific materials, systems and methods

We require information from authors about some types of materials, experimental systems and methods used in many studies. Here, indicate whether each material, system or method listed is relevant to your study. If you are not sure if a list item applies to your research, read the appropriate section before selecting a response.

Materials & experimental systems

n/a	Involved in the study	
☒	☐	Antibodies
☒	☐	Eukaryotic cell lines
☒	☐	Palaeontology and archaeology
☐	☒	Animals and other organisms
☒	☐	Human research participants
☒	☐	Clinical data
☒	☐	Dual use research of concern

Methods

n/a	Involved in the study	
☒	☐	ChIP-seq
☒	☐	Flow cytometry
☒	☐	MRI-based neuroimaging

Animals and other organisms

Policy information about studies involving animals; ARRIVE guidelines recommended for reporting animal research

Laboratory animals	Mice (C57BLJ6; female; 6- and 68- week, n=6 biologically independent samples for each group) were used in this study. All animal experiments were approved by the Institutional Animal Care and Use Committee (IACUC) of Interdisciplinary Research Center on Biology and Chemistry, Shanghai Institute of Organic Chemistry, Chinese Academy of Sciences, and complied with all relevant ethical regulations. The 6-week and 68-week old female mice were purchased from Xiamen University Laboratory Animal Center (Xiamen, China). All mice were maintained on a 12-h light/dark cycle at room temperature (24 ± 2 °C) with constant humidity (40 ± 15%).
Wild animals	The study did not involve any wild animals.
Field-collected samples	The study did not involve samples collected from the filed.

Ethics oversight	All animal experiments were approved by the Institutional Animal Care and Use Committee (IACUC) of Interdisciplinary Research Center on Biology and Chemistry, Shanghai Institute of Organic Chemistry, Chinese Academy of Sciences, and complied with all relevant ethical regulations.

Note that full information on the approval of the study protocol must also be provided in the manuscript.
